# 《胸部恶性肿瘤围术期静脉血栓栓塞症预防中国专家共识（2018版）》解读之D-二聚体篇

**DOI:** 10.3779/j.issn.1009-3419.2019.12.05

**Published:** 2019-12-20

**Authors:** 青山 陈, 智荣 章, 红红 董, 劲柏 苗, 辉 李

**Affiliations:** 100020 北京，首都医科大学附属北京朝阳医院胸外科 Department of Thoracic Surgery, Beijing Chaoyang Hospital, Capital Medical University, Beijing 100020, China

**Keywords:** 胸部恶性肿瘤, 静脉血栓栓塞症, D-二聚体, 临床意义, Thoracic cancer, Venous thromboembolism, D-dimer, Clinical significance

## Abstract

胸部恶性肿瘤患者围术期发生静脉血栓栓塞症（venous thromboembolism, VTE）风险较高，VTE的发生将影响患者的预后、远期生活质量，严重者甚至致死。为了加强胸外科医生对胸部恶性肿瘤患者围术期VTE的了解和重视，中国胸外科静脉血栓栓塞症研究协作组发布了《胸部恶性肿瘤围术期静脉血栓栓塞症预防中国专家共识（2018版）》。本文针对共识中D-二聚体在VTE中的诊断价值及风险预测价值进行详细解读，并对其他生物标记物在肿瘤患者VTE的作用做简要拓展，以加深胸外科医生对D-二聚体在VTE中临床意义的理解。

## Abstract (en)

The risk of perioperative venous thromboembolism (VTE) is pretty high in thoracic cancer patients. Perioperative VTE influences the recovery of patients after operation and quality of life in the future, even seriously leading to death. To strengthen the knowledge and attention of thoracic surgeons on perioperative VTE in thoracic cancer patients, China National Research Collaborative Group on VTE in Thoracic Surgery released the edition of VTE prophalaxis in thoracic cancer patients: Chinese experts consensus in 2018. This article is to interpret the diagnostic value and risk prediction value of D-dimer in VTE in detail, and briefly introduce the role of other biomarkers in VTE of tumor patients. The consensus interpretation aims to deepen the understanding of thoracic surgeons on the clinical significance of D-dimer in VTE.

肺癌和食管癌是我国两类最常见的胸部恶性肿瘤，研究已证实肺癌和食管癌均是发生静脉血栓栓塞症（venous thromboembolism, VTE）的高危因素，特别是围术期的发病率较高，且围术期VTE的发生将增加死亡风险。2018年由中国静脉血栓栓塞症研究协作组主导，在《中国肺癌杂志》上发表了《胸部恶性肿瘤围术期静脉血栓栓塞症预防中国专家共识（2018版）》^[1]^。该文对胸部恶性肿瘤VTE的流行病学及我国胸外科VTE的防治现状进行了介绍，对VTE的风险因素及评估方法、诊断、防治措施进行了系统性总结，最后对胸部恶性肿瘤VTE的防治提出了指导性建议。

本文将对D-二聚体在静脉血栓诊断中的作用进行介绍，重点对D-二聚体在胸部恶性肿瘤VTE诊断中的特殊性及临床应用进行总结，旨在加强胸外科医生对D-二聚体临床意义及应用的理解，加深对胸部恶性肿瘤VTE的认识。

## 1 D-二聚体在临床VTE中的诊断价值

### 1.1 D-二聚体检测可以辅助VTE诊断的排除

正常的D-二聚体水平与较低的临床VTE可能性评估相结合能高效地除外VTE，减少不必要的临床检查以及避免不必要的抗凝治疗。

D-二聚体是纤维蛋白的最小降解产物，由纤维蛋白酶蛋白水解纤维蛋白产生，是纤溶系统激活的敏感标记，已成为最有效的纤溶标记物^[2, 3]^。在VTE事件中，D-二聚体水平会异常升高，通过D-二聚体水平的测定能够辅助VTE的诊断^[4-6]^。D-二聚体的检测方法有酶联免疫吸附测定法（enzyme-linked immunosorbent assay, ELISA）、乳胶凝集试验法及全血凝集试验法，但ELISA法相对于另外两种检测方法，有更高的灵敏度及阴性预测价值，检测结果更客观，且不受检测人员因素干扰^[7]^。目前ELISA法被广泛用于临床D-二聚体的测定。

对于临床疑似存在VTE患者，D-二聚体水平与临床VTE可能性评估量表（常用为Wells评分量表）将提高诊断效率，同时能够避免不必要的临床检查与抗凝治疗。D-二聚体（ELISA法）在诊断VTE时具有较高的灵敏度（> 95%），但特异度较差^[8, 9]^。当患者临床发生深静脉血栓（deep vein thrombosis, DVT）可能性较低时（Wells评分≤1分），若此时患者D-二聚体水平正常（< 500 μg/L）则可明确除外DVT，无需进行更多的超声或者影像学检查，且这些患者在接下来3个月内发生VTE的风险非常低（< 1%）^[10, 11]^；当患者临床考虑发生肺栓塞（pulmonary embolism, PE）的可能性低时（Wells评分≤4分或者采用简化Wells评分量表评分 < 2分），结合正常水平D-二聚体也能够安全除外PE^[12-14]^，这类患者在随后3个月内发生PE的风险同样较低（< 3%），无需进行不必要的抗凝^[15]^。

### 1.2 D-二聚体在诊断胸部恶性肿瘤VTE中诊断价值受限

尽管D-二聚体水平的检测能够高效地辅助VTE的除外诊断，但在恶性肿瘤患者疑似VTE时的除外诊断中，D-二聚体水平与临床VTE可能性评估相结合的诊断策略在VTE的除外诊断中可靠性和效率均有所限制。

胸部恶性肿瘤常呈高凝状态，这与恶性肿瘤自身的特点有关^[16]^，即使无VTE发生，这部分人群的D-二聚体水平往往呈高水平状态，这导致了D-二聚体处于正常水平的患者比例较低^[17]^；其次，肿瘤常见于高龄患者，肿瘤和非肿瘤原因造成其住院频率较高，加上相关的治疗，这些因素均能影响D-二聚体水平。对于恶性肿瘤患者，当利用D-二聚体辅助VTE的除外诊断时其可靠性和效率受到影响^[18]^。

D-二聚体对于恶性肿瘤患者疑似DVT的诊断价值目前还有争议。在一项利用Wells评分量表结合D-二聚体水平诊断DVT的*meta*分析中，该项研究包含了多个地区的13项研究，涉及10, 002例患者，当选择Wells评分≤1分结合正常水平D-二聚体（< 500 μg/L）作为排除DVT的诊断标准时，对其中的恶性肿瘤患者分析发现，仅有9.1%的患者考虑除外DVT，而非肿瘤患者中则有23%-43%的患者考虑除外DVT；此外这些考虑除外DVT的肿瘤患者中，在接下来3个月的随访中DVT的发生率为2.2%，已经超过了静脉造影检查的漏诊率（2%）。因此对于恶性肿瘤患者采用此诊断策略除外DVT时效率低，同时安全性也不可靠^[10]^。

而对于恶性肿瘤患者在考虑PE时，当Wells评分≤4分，同时D-二聚体 < 500 μg/L作为排除PE的诊断标准时，研究发现该诊断策略是安全的，但是能够安全除外的非PE患者比例过低，不足10%^[18]^。之后关于Wells评分结合D-二聚体除外PE的*meta*分析也得到同样的结论，能安全除外的非PE肿瘤患者占9.1%，3个月随访中PE发生率为2.6%，小于肺动脉造影漏诊率的3%^[15]^。

即便该诊断策略对于肿瘤患者的VTE诊断有诸多缺陷，也不能否定其临床价值，它能够快速、便捷地为临床医师提供首要的诊断信息，能够辅助判断患者是需要进一步检查还是继续加强随访。对于安全性而言，希望能通过密切随访避免漏诊，对于效率而言，则应当选择更为合适的D-二聚体正常临界值。

### 1.3 胸部恶性肿瘤患者应依据年龄选择不同的D-二聚体正常临界值

为了提高在恶性肿瘤患者疑似VTE时的除外诊断效率，应当根据年龄选择合适的D-二聚体正常临界值。

研究^[19, 20]^发现D-二聚体水平随着年龄增长而升高，这将导致D-二聚体在辅助诊断恶性肿瘤患者VTE时特异性及效率降低。Harper等^[21]^在针对临床考虑静脉血栓患者的调查中发现D-二聚体水平在不同年龄阶段并非一致，而是随着年龄增长而上升。在40岁以下、40岁-60岁、61岁-80岁及80岁以上不同年龄阶段患者D-二聚体平均水平分别为294 μg/L、387 μg/L、854 μg/L及1, 397 μg/L。若仍以常规D二聚体正常临界值（500 μg/L）为标准，那么在40岁以下患者中特异性为70.5%，而61岁-80岁患者及80岁以上患者的特异性则分别仅为25.3%及4.5%。随后一项多中心回顾分析及meta分析也证实^[22-24]^，在50岁以上患者中选择（年龄×10 ng/mL）作为D-二聚体正常临界值上限时，其安全性及灵敏性未受到明显影响，特异性则至少提高了10%。

肿瘤患者除了老龄患者较多外，高凝状态常伴有D-二聚体水平上升，若仍以常规D-二聚体临界值（500 μg/L）作为诊断标准，诊断的特异性及效率会明显降低。在D-二聚体与Wells评分相结合诊断PE的*meta*分析中，对于考虑PE的恶性肿瘤患者，当分别选择常规D-二聚体临界值（500 μg/L）与年龄相关的D-二聚体临界值时（> 50岁时，年龄×10 μg/L）与Wells评分相结合两种不同诊断策略时发现，除外PE的患者比例由9.1%增加到13.1%，效率有所增加，安全性并未受到影响^[15]^。但是我们应当注意的是，即便根据年龄调整了D-二聚体的临界值，诊断效率仍旧较低。因此仍有不少非PE肿瘤患者无法避免额外的影像学检查。目前对于临床考虑DVT的肿瘤患者，还缺乏常规D-二聚体临界值与年龄调整后D-二聚体临界值相比较的*meta*分析，可以猜测D-二聚体临界值根据年龄调整后，诊断效率会有所增加，但增加幅度不会特别大。

当根据年龄选择D-二聚体临界值时（> 50岁，年龄×10 μg/L），对于疑似VTE的肿瘤患者，除外VTE的诊断效率升高，但仍旧有限。一方面可能是由于疑似PE的肿瘤患者D-二聚体水平远高于年龄×10 μg/L，当年龄 > 50岁时，选择年龄×10 μg/L作为D-二聚体临界值并非最优，可能选择年龄×11 μg/L、年龄×12 μg/L或者年龄×13 μg/L等作为D-二聚体临界值更为合适，这需要更多针对肿瘤患者的研究甚至更多仅针对胸部肿瘤患者的研究来确定合适的D-二聚体临界值。另一方面可能是由于低年龄组增加的效率过低影响了总体的诊断效率，是否选择更高的年龄作为改变D-二聚体临界值的分界也值得思考。

## 2 D-二聚体在VTE中的风险预测价值

### 2.1 D-二聚体在近期未予治疗的肿瘤患者中发挥VTE风险预测作用

对于近期未予以任何抗肿瘤治疗的患者，D-二聚体水平升高预示着VTE发生的风险增加。

在一项长期、多中心队列研究中发现患者较高的D-二聚体水平与随后静脉血栓形成的风险增加相关^[25]^。Ay等^[26]^进一步在对包括119例肺癌患者在内的实体肿瘤患者VTE风险预测研究，这些研究对象均为新发肿瘤患者或者处于进展期的肿瘤患者，但过去2周内未进行任何放疗或者手术，且过去3个月内未进行任何化疗，研究对象进入研究之初便检测D-二聚体及凝血酶原片段1+2（fragment 1+2, F1+2）水平。结果显示在包括D-二聚体、F1+2水平在VTE发生的患者中明显升高；*Cox*多因素回归分析发现D-二聚体水平升高及F1+2水平升高将分别导致患者VTE发生风险增加1.8倍及2倍，若患者D-二聚体与F1+2水平同时升高则VTE发生风险将增加3.6倍。对研究对象随访6个月后累积VTE风险进行*K*-*M*（*Kaplan*-*Meier*）分析，肿瘤患者发生VET风险在D-二聚体及F1+2均升高的肿瘤患者中最高，而D-二聚体和F1+2未升高患者发生VTE的风险较低（15.2% *vs* 5.0%, *P* < 0.001）^[26]^。因此对于胸部恶性肿瘤患者，当D-二聚体与F1+2水平均升高时，予以抗凝预防治疗将可能取得较大临床益处。但目前尚缺乏关于胸部恶性肿瘤中D-二聚体预测VTE风险的大规模研究，在胸部恶性肿瘤患者中升高的D-二聚体能发挥预测VTE的风险如何尚不清楚；同时对这些患者予以预防抗凝治疗所得到临床益处也不确定，需要进一步研究证实。

### 2.2 D-二聚体在肺癌化疗后VTE中具有风险预测作用

目前指南不建议门诊化疗患者常规抗凝^[27]^，但化疗本身能增加肿瘤患者发生VTE的风险。已有研究发现D-二聚体水平在肺癌化疗后VTE中具有风险分层作用。

在一项关于前瞻性、单中心研究^[28]^中发现肿瘤（包括肺癌）患者化疗前D-二聚体水平与化疗后VTE发生相关，D-二聚体水平 > 650 μg/L的肿瘤组中VTE事件发生率为22%，D-二聚体水平 < 650 μg/L的肿瘤组中VTE事件发生率仅为2.4%（*P*=0.003）。但此次研究有关肺癌的研究对象仅为20例（16.2%），不足以体现D-二聚体在肺癌化疗后VTE中的预测价值。之后有研究^[29]^进一步对108例已进行化疗的肺癌患者D-二聚体水平回顾性分析，所有患者进行1期以铂类为主的化疗方案，根据*Khorana*风险评分后均被列为中危组。分析发现当取1, 500 μg/L作为D-二聚体正常临界值时诊断的准确性最高，此时准确性为70.4%，特异性为68.5%，灵敏性为81.3%。对研究对象未发生VTE的间期进行K-M分析发现D-二聚体高于正常水平的患者相比于正常水平患者，从研究开始到发生VTE事件的间隔更短（*P*=0.000, 1）。多因素Cox回归分析则发现高水平D-二聚体患者相比于正常水平患者，发生VTE的风险增加了11倍（*P*=0.001）^[29]^。可以看到当*Khorana*风险评分结合D-二聚体水平时，可以更准确地筛选出那些VTE风险较高的患者，并能针对性予以抗凝预防治疗。但D-二聚体水平在肺癌化疗后VTE风险预测中是否有如此大的作用还有待更大样本、更多研究证实。

### 2.3 D-二聚体在肺癌术后VTE中的风险预测作用

外科手术也是VTE的风险因素之一，术后VTE的总发生率约为0.8%-1.6%^[30, 31]^，然而肺癌术后VTE发生率更高，约为0.2%-19%^[32]^。目前关于D-二聚体对肺术后VTE风险预测意义的研究较少。在关于肺癌术后VTE发生的风险因子研究中发现，术前高水平D-二聚体是术后VTE独立的风险因子^[33]^。在一项包括111例肺良恶性肿瘤术后VTE事件的单中心、前瞻性研究^[34]^中也有类似的发现，发生VTE的患者术前（*P*=0.007）、术后第1天（*P*=0.005）、术后第3天（*P*=0.002）D-二聚体水平均明显高于未发生VTE的患者，研究者建议术前D-二聚体水平较高的患者应予以抗凝预防治疗。但目前还需大样本、多中心研究进一步证实，且术前D-二聚体高水平患者究竟采取哪些特殊的血栓预防措施也有待研究。

## 3 生物标志物在胸部肿瘤患者VTE中的临床意义

除D-二聚体能辅助预测VTE风险外，许多生物标志物也能帮助预测肿瘤VTE发生风险^[35]^。可溶性P-选择素（soluble P-selectin, sP-sel）是反映体内血小板活化的标志物^[36]^，有研究^[37-39]^已发现高水平sP-sel与VTE发生相关；在一项前瞻性研究^[40]^中也发现sP-sel是VTE复发的危险因子；之后的临床证据^[41]^也表明sP-sel可能是肿瘤患者VTE独立的风险预测因子。但是部分生物标记物在预测胸部肿瘤相关的VTE发生风险时还在一定争议与缺陷。先前的研究^[42]^表明，炎症介质的水平是静脉血栓疾病的危险指标，肿瘤坏死因子α（tumor necrosis factor α, TNF-α）、白介素6（interleukin 6, IL-6）和白介素8（interleukin 8, IL-8）水平是静脉血栓形成的风险决定因素；然而在关于白介素与肿瘤患者VTE之间关系的研究中发现，虽然IL-1β、IL-6水平与胰腺癌VTE发生之间有一定关系，但是并未发现白介素与胸部肿瘤患者VTE之间的关联^[43]^。由凋亡或活化细胞产生的微粒（microparticles, MPs）也具有激活凝血的功能，与未发生静脉血栓的肿瘤患者相比，发生静脉血栓的肿瘤患者MPs水平是较高的^[44]^；多项研究^[45, 46]^发现升高水平的MPs能预测血栓发生风险，但是也有研究^[47]^发现MPs在血栓发生中并没有预测价值，这种差异可能是由于MPs的检测方法标准差异造成的^[48]^。除外以上这些生物标志物，还有血小板数量、白细胞数量、血小板/中性粒细胞比率、中性粒细胞/淋巴细胞比率以及一些生化参数均发现与肿瘤相关VTE有一定关系^[35]^，但这些生物标记物与D-二聚体一样均缺乏较高的特异性，需要与多种因素相结合提高风险预测作用。

## 4 小结

D-二聚体在疑似VTE诊断中敏感性较高，D-二聚体水平正常，且临床Wells评分VTE可能性低时，能安全地除外VTE可能，无需再进行更多的影像学检查。但对于疑似VTE胸部恶性肿瘤患者时，该诊断策略诊断效率有限同时也并非绝对安全可靠，但能为临床医师提供一定的诊断信息，临床医师应该对疑似VTE恶性肿瘤患者加强随访，同时适时采取进一步检查以除外VTE。同时在胸部恶性肿瘤患者中，D-二聚体也能在VTE事件中发挥一定的风险预测作用，高水平D-二聚体将提醒我们肿瘤患者在治疗期间VTE发生风险增加，可以针对性采取抗凝预防措施，降低VTE发生的可能。除D-二聚体外，目前也有许多生物标志物能够辅助预测胸部恶性肿瘤VTE的发生风险，但这些生物标记物与D-二聚体一样均缺乏较高的特异性，一方面需要我们将各种不同的VTE风险评估量表与这些众多具有风险预测作用的生物标志物相结合综合分析；另一方面也提醒我们可以利用肿瘤细胞所介导参与的凝血及纤溶系统活化，寻找诊断肿瘤患者VTE特异性更高的指标。
